# Analysis of the Accuracy and Robustness of the Leap Motion Controller

**DOI:** 10.3390/s130506380

**Published:** 2013-05-14

**Authors:** Frank Weichert, Daniel Bachmann, Bartholomäus Rudak, Denis Fisseler

**Affiliations:** Department of Computer Science VII, Technical University Dortmund, Dortmund 44221, Germany; E-Mails: daniel.bachmann@tu-dortmund.de (D.B.); rudak@ls7.cs.tu-dortmund.de (B.R.); denis.fisseler@tu-dortmund.de (D.F.)

**Keywords:** leap motion controller, accuracy, robustness, measurement precision

## Abstract

The Leap Motion Controller is a new device for hand gesture controlled user interfaces with declared sub-millimeter accuracy. However, up to this point its capabilities in real environments have not been analyzed. Therefore, this paper presents a first study of a Leap Motion Controller. The main focus of attention is on the evaluation of the accuracy and repeatability. For an appropriate evaluation, a novel experimental setup was developed making use of an industrial robot with a reference pen allowing a position accuracy of 0.2 mm. Thereby, a deviation between a desired 3D position and the average measured positions below 0.2 mm has been obtained for static setups and of 1.2 mm for dynamic setups. Using the conclusion of this analysis can improve the development of applications for the Leap Motion controller in the field of Human-Computer Interaction.

## 1. Introduction

In the last few years, different optical sensors, which allow the acquisition of 3D objects, have been developed. Concurrently with the appearance of the new sensors, the number of potential applications vastly increases. Applications benefit especially from the increasing accuracy and robustness of 3D sensors [1] and a drop in prices. Applications for 3D sensors include industrial tasks, people and object tracking, motion analysis, character animation, 3D scene reconstruction and gesture-based user interfaces [2]. These applications have different requirements in terms of resolution, speed, distance and target characteristics. Particularly with regard to gesture-based user interfaces, the accuracy of the sensor is a challenging task. Consumer-grade sensors offer only limited positioning accuracy. An analysis of the Kinect controller shows a standard deviation in depth accuracy of approximately 1.5 cm [1]. The evaluation of the accuracy of optical sensors is the subject of current research and scientific discussion [3].

The Leap Motion controller introduces a new gesture and position tracking system with sub-millimeter accuracy. In contrast to standard multi-touch solutions, this above-surface sensor is discussed for use in realistic stereo 3D interaction systems [4], especially concerning direct selection of stereoscopically displayed objects [5]. To the knowledge of the authors, the controller's capabilities in realistic environments have not been analyzed. Therefore, this paper presents a first study of the Leap Motion controller's accuracy and repeatability abilities. The major contributions of this paper are:

- Analysis of the accuracy and robustness of the Leap Motion controller.
- Specification of an objective test setup for 3D sensors using an industrial robot system.
- Definition of quality criteria considering industrial specifications.

The paper is structured as follows. After this introduction, related work is presented. The principles of the experimental environment are introduced in Section 3. Based on the described setup, experiments designed to evaluate the sensor's accuracy and repeatability are expounded in Section 4. In Section 5 the experimental results are analyzed, which leads to the conclusions represented in Section 6.

## 2. Related Work

In the following, an overview relating to existing optical *3D* sensors and the calibration techniques is presented followed by a categorization of the Leap Motion controller and the motivation of the new calibration setup.

The operating principle of the measurement of *optical 3D sensors* can be, in principle, divided into the following mechanisms: Structured Light, Time of Flight and Stereo-Vision. Structured light sensors analyze the deformation (warping) of a known pattern onto an unknown surface to determine the three-dimensional shape [6]—representative examples include Microsoft's Kinect sensor (Kinect For Windows, http://www.microsoft.com/en-us/kinectforwindows) and Asus Xtion Live (Asus Xtion Pro Live, http://www.asus.de/Multimedia/Motion_Sensor/Xtion_PRO). For a generalized overview of comparisons between the mechanisms, see, e.g. [7]. The Time of Flight (TOF) 3D cameras are based on the well-known time of flight principle [8]. Additionally, there is a differentiation between PMD (Photonic Mixer Device) and laser sensors. A PMD sensor (e.g., Swissranger 4000 (Mesa Imaging, http://www.mesa-imaging.ch), PMDVision CamCube 3.0 (PMDVision, http://www.pmdtec.com)) measures the distance to an object by emitting modulated infrared light and determining the phase shift between the emitted and reflected light. In case of a laser sensor (e.g., Laser Sick LMS511 (Laser Sick LMS511, http://www.sick.com/group/en/home/products/product_news/industrial instrumentation/pages/bulkscan_laser_volume_flowmeter.aspx)) the distance to an object is measured by emitting pulsed laser beams and determining the time the reflected light needs to travel back to the sensor. Stereo Vision cameras (e.g., the Bumblebee 2 sensor (Point Grey Research, http://www.ptgrey.com/products/bumblebee2)) consist of two optical 2D cameras with known extrinsic parameters. The concept of determining the depth in the scene is based on searching correspondence points in both 2D images [9]. Optical tracking systems (e.g., [10]) use the raw data (n-dimensional point clouds) of optical 3D sensors in order to detect the position of predefined markers in the Cartesian space of the viewed scene.

The evaluation and calibration of optical sensors are based on reference objects with known dimensions and positions in Cartesian space. An overview of calibration methods of TOF sensors can be found in [11]. Weingarten [12] captures a planar wall in different manually chosen distances to the used PMD camera and derives a correction function for the systematic distance error, which is optimized by Rapp [13] through precise repositioning of the PMD camera by a linear axis. The irregularity in the planarity of the wall, as reference object for the calibration process of the Swissranger SR400 PMD camera, is compensated by Chiabrando *et al.* [14] through capturing the wall with a high resolution laser scanner. A high precision laser scanner is also used by Khoshelam [1] in order to compare the deviations of captured reference objects with the point cloud generated with the structured light based Kinect camera. Stoyanov *et al.* [3] generate ground truth scans of arbitrary reference objects with a laser scanner SICK LMS-200 in order to compare them with point clouds provided by different range sensors for indoor environments. A comparison of the relative accuracy between a mechanical and an optical position tracker for image-guided neurosurgery is presented by Rohling *et al.* [15]. A reference aluminum block with precisely drilled holes, which are detected by the mechanical and optical position tracker, serves as ground truth. Koivukangas *et al.* [16] use a specially designed accuracy assessment phantom, a cube with high precision set assessment point, in order to evaluate the accuracy of optical tracking systems used in surgical area.

**Figure 1.**
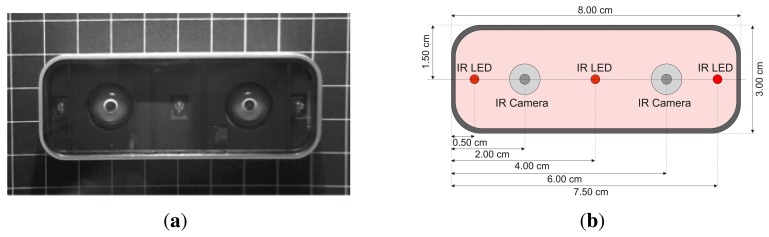
Visualization of a (**a**) Real (using Infrared Imaging) and (**b**) Schematic View of Leap Motion Controller.

The Leap Motion controller in conjunction with the current API (Application Programmer Interface) delivers positions in Cartesian space of predefined objects like finger tips, pen tip, *etc.* The delivered positions are relative to the Leap Motion controller's center point, which is located at the position of the second, centered infrared emitter (*cf*. Section 3.1). As illustrated in Figure 1, the controller consists of three IR (Infrared Light) emitters and two IR cameras. Hence, the Leap Motion can be categorized into optical tracking systems based on Stereo Vision. Because of the missing point cloud of the scene and the predefined detectable objects, traditional calibration techniques are not suitable for the Leap Motion. Nevertheless, a precise reference system is needed in order to evaluate the accuracy and repeatability of the Leap Motion controller. Industrial Robots support the ability of fixing different tools to their TCP (Tool Control Point) and exhibit high precision in sub-millimeter range. Consequently, industrial robots can act as high precision reference systems during the evaluation of the Leap Motion.

The novel calibration setup as described in Section 3.2 uses an industrial robot with a reference pen, which is defined as a robot tool and detected by the Leap Motion controller. The goal of the novel experimental setup is to allow the tracking of the reference pen tip simultaneously by the robot and the Leap Motion controller.

## 3. Experimental Environment

Taking into account that the majority of applications for the Leap Motion controller are gesture-based user interfaces, the achievable accuracy of measurement of the motion of a human hand is the most relevant factor, which is essentially affected by the so-called tremor. Tremor is defined as an involuntary and approximately rhythmic movement of muscles. Depending on the human age, the tremor amplitude varies between 0.4 mm ± 0.2 mm for young individuals and 1.1mm ± 0.6mm for old individuals [17,18]. Hence, in order to evaluate the Leap Motion controller with regard to human gesture-based user interfaces, a reference system with an accuracy below the human tremor—below 0.2 mm—must be established. To meet these demands, an industrial robot, Kuka Robot KR 125/3, which provides a repeatable accuracy of less than 0.2 mm, was chosen. The test setup consists of the Leap Motion controller (Section 3.1) and a robot cell comprising an industrial robot (Section 3.2).

### 3.1. Leap Motion Controller

The Leap Motion controller is a new consumer-grade sensor developed by Leap Motion (Leap Motion, http://www.leapmotion.com). It is primarily designed for hand gesture and finger position detection in interactive software applications. Because of current patent pending, only insufficient information on the underlying software's geometrical or mathematical frameworks is available. Nevertheless, first impressions of position detection capabilities are promising (Section 5). Thus, the presented analysis does not focus on the controller's technical details. A detailed analysis of the sensor's accuracy and repeatability will be presented. Figure 1 shows a schematic view of the controller's hardware setup. Besides three infrared emitters the device incorporates two CCD cameras (cf. Figure 1(a)). As stated by the manufacturer, the sensor's accuracy in fingertip position detection is approximately 0.01 mm.

By designing a new measurement setup based on an industrial robot, the controller's repeatability and accuracy are reviewed and evaluated with respect to the Leap Motion controller's gesture and motion detection capabilities.

### 3.2. Measurement Setup

**Figure 2.**
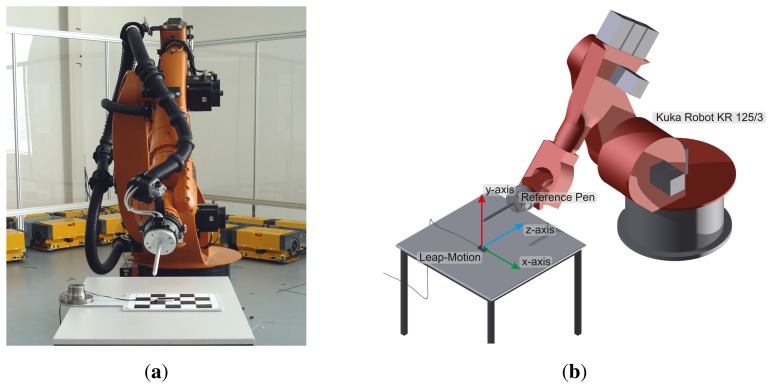
Visualization of the Robot Cell Consisting of the Leap Motion Controller, an Industrial Robot (Kuka Robot KR 125/3) with a Reference Pen: (**a**) Front View and (**b**) Schematic View with a Coordinate System.

A modified robot cell is utilized as the measurement setup consisting of a Leap Motion controller, an industrial robot (Kuka (KUKA Roboter GmbH, http://www.kuka-robotics.com) KR 125/3) and a reference pen as visualized in Figure 2. The Leap Motion controller is fixed on a plane in the range of the robot TCP (Tool Control Point) and the reference pen is attached to the robot tool [19]. By establishing a fixed known relationship of the pen tip and the TCP and defining the pen as a new robot tool, an indirect reference through the robot kinematics to the world coordinate system of the robot is created [20]. Hence, two static coordinate systems are linked through the pen tip point, the world coordinate system of the robot and of the Leap sensor.

A laptop computer, Intel^®^ Core™2 CPU 3.06 GHz with 8 GB RAM, with the developed evaluation software serves as a central control device between the Leap Motion controller and the robot. The measurement process begins with defining an ROI (Region Of Interest) that represents the subspace of the reference pen tip positions in the robot coordinate system so that each pen tip position in the subspace lies in the range of the sensor. Then a set of positions in the ROI is defined representing discrete measurement points according to the particular test case. In order to evaluate the influence of the dimension of the reference pen on the measurements, reference pens with different diameters (*d*_1_= 3 mm, *d*_2_ = 4 mm, *d*_3_ = 5 mm, *d*_4_ = 6 mm, *d*_5_ = 8 mm, *d*_6_ = 10 mm) were used.

### 3.3. Metrology

The robot cell builds the metrology system [21] of the mandatory measurements. The analyzed parameters related to the sensors are accuracy and repeatability. Accuracy is the ability of a 3D sensor to determine a desired position in 3D space. Repeatability is the ability of a sensor to locate the same position in every measurement repetition. The analysis of the accuracy and repeatability tests was performed in accordance to ISO 9283 [22] standard, which is primarily used for industrial robots.

In the following, mathematical definitions of the aforementioned parameters are introduced. The reference positions are denoted by ${\text{p}}^{\left[i\right]}={\left(p_{x}^{\left[i\right]},p_{y}^{\left[i\right]},p_{z}^{\left[i\right]}\right)}^{T}\in {\mathbb{R}}^{3}$ for *i* = 1,…, *P* and the corresponding measured positions by ${\tilde{\text{p}}}^{\left[i,j\right]}={\left({\tilde{p}}_{x}^{\left[i,j\right]},{\tilde{p}}_{y}^{\left[i,j\right]},{\tilde{p}}_{z}^{\left[i,j\right]}\right)}^{T}$ for *j* = 1,…, *R*. Each measurement is performed on *P* positions. Each position is repeatedly measured over a constant time interval with frequency *R*. The accuracy *Acc_i_* for a desired 3D position ${\text{p}}^{[i]}={\left(p_{x}^{\left[i\right]},p_{y}^{\left[i\right]},p_{z}^{\left[i\right]}\right)}^{T}$ is calculated by

(1) $$Acc_{i}=\sqrt{{\left(m_{{\tilde{p}}_{x}^{\left[i,j\right]}}-p_{x}^{\left[i\right]}\right)}^{2}+{\left(m_{{\tilde{p}}_{y}^{\left[i,j\right]}}-p_{y}^{\left[i\right]}\right)}^{2}+{\left(m_{{\tilde{p}}_{z}^{\left[i,j\right]}}-p_{z}^{\left[i\right]}\right)}^{2}}$$

with

(2) $$m_{{\tilde{p}}_{x}^{\left[i,j\right]}}=\frac{1}{R}\sum_{j=1}^{R}{\tilde{p}}_{x}^{\left[i,j\right]},m_{{\tilde{p}}_{y}^{\left[i,j\right]}}=\frac{1}{R}\sum_{j=1}^{R}{\tilde{p}}_{y}^{\left[i,j\right]},m_{{\tilde{p}}_{z}^{\left[i,j\right]}}=\frac{1}{R}\sum_{j=1}^{R}{\tilde{p}}_{z}^{\left[i,j\right]}$$

the arithmetic mean of the positions acquired by the *R* measurements of **p**^[*i*]^. Repeatability is calculated by

(3) $${\text{Rep}}_{i}=m_{D_{i}}+3S_{i}$$

with

(4) $$m_{D_{i}}=\frac{1}{R}\sum_{j=1}^{R}D_{i,j},\;D_{i,j}=\sqrt{{\left({\tilde{p}}_{x}^{\left[i,j\right]}-m_{{\tilde{p}}_{x}^{\left[i,j\right]}}\right)}^{2}+{\left({\tilde{p}}_{y}^{\left[i,j\right]}-m_{{\tilde{p}}_{y}^{\left[i,j\right]}}\right)}^{2}+{\left({\tilde{p}}_{z}^{\left[i,j\right]}-m_{{\tilde{p}}_{z}^{\left[i,j\right]}}\right)}^{2}}$$

the variance of the measured positions and

(5) $$S_{i}=\sqrt{\frac{\sum_{j=1}^{R}{\left(D_{i,j}-m_{D_{i}}\right)}^{2}}{R-1}}$$

the corresponding standard deviation. The arithmetic mean of the accuracy over all desired 3D positions **p**^[*i*]^, *i* = 1, …, *P* is $\text{Acc}=\frac{1}{P}\sum_{i=1}^{P}Acc_{i}$ and for the repeatability $\text{Rep}=\frac{1}{P}\sum_{i=1}^{P}{\text{Rep}}_{i}$.

In the following, experiments are represented in order to analyze the sensor's accuracy and repeatability in static and dynamic scenarios.

## 4. Experiments

For the purpose of sensor evaluation, test cases are defined that take the aforementioned metrological aspects and the experimental test setup with the modified robot cell into account. In order to test the sensor's accuracy and repeatability, a static scenario is chosen that allows the measurement of the maximum deviation from a known position over time. A dynamic scenario is used to test the ability of the sensor to acquire the accurate position of a moving object.

The following tests were conducted considering the metrology calibration approaches [23]:

- Positioning test probe methods (static cases)
- Path drawing methods (dynamic cases)

**Figure 3.**
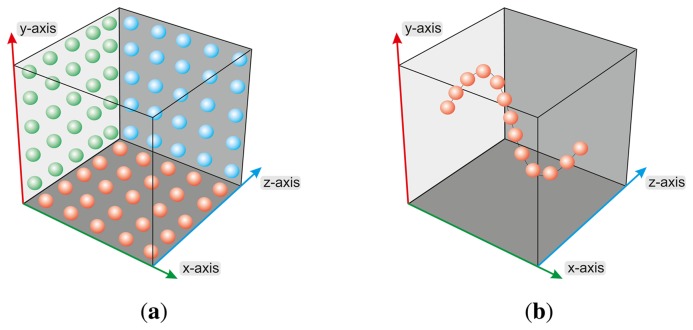
Visualization of the Basic Test Cases: (**a**) Positions in the xy-, xz- and yz-Plane and (**b**) Positions on a Sinus Function Within the xy-Plane.

The basic test cases focus on the evaluation of the accuracy of the reference pen tip moving to positions on a regular grid of a plane (xy-, xz- and yz-plane) and moving to discrete positions on a path, for example along the particular axes (x-, y- and z-axis) of the sensor coordinate system and on a sinus function within the xy-plane, as illustrated in Figure 3. During the measurement process, the reference pen remains in a static pose bowed over the sensor, analogous to navigating with a human finger tip. The measurement process proceeds in a loop while in each cycle the robot takes a particular position p^[^*^i^*^]^ as a static pose. The process ends when all *P* positions are measured. In each cycle, after reaching the new robot position, a series of *R* sensor measurements of the reference pen tip are captured. In order to avoid mechanical oscillation, the velocity of the robot is reduced to a minimum in combination with a time wait slot of 250 ms for the sensor measurements after reaching each new robot position. To avoid numerical deviations due to inconsistent lighting or ambient temperature conditions, the test setup was build with constant ambient temperature of 23 °C and constant luminous flux of approximately 250 lx. The result of the measurement process is a set of *P* correspondence tuples consisting of positions **p** representing the Cartesian coordinates of the reference pen tip in the robot coordinate system and the corresponding discrete sensor measurements of the reference pen tip in the Cartesian coordinates of the Leap Motion controller.

The following test cases distinguish between static and dynamic scenarios. Within the static scenarios, the reference pens with different diameters (*d*_1_= 3 mm, *d_2_* = 4 mm, *d*_3_= 5 mm, *d*_4_ = 6 mm, *d*_5_= 8 mm, *d_6_* = 10 mm) are moved to a desired position by the robot and remain unmoved, in a fixed parking position, during the measurement process with 5,000 measurements in each case. Furthermore, a long time measurement of 240 minutes is performed. During the dynamic measurement scenarios, the robot approaches different positions of the pen tip in the pre-limited Cartesian space. The reference pen is moved on one hand on linear paths of 200 mm along the xy-, xz- and yz-planes, centered on the center point of the Leap Motion controller, and on the other hand on a path along a sinus function in the xy-plane with a support of 50 mm centered on the controller's center point.

## 5. Results

Based on the denned test cases in Section 4, the presentation of the measurement results is subdivided into static cases Section 5.1 and dynamic cases Section 5.2.

### 5.1. Static Test Cases

**Figure 4.**
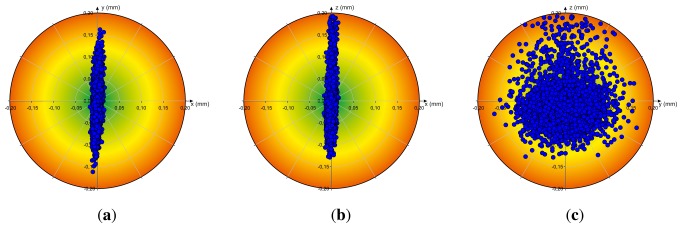
Analysis of the Accuracy and the Repeatability: Deviation between a desired 3D Position and the Measured Positions for a Static Position, (**a**) xy-Variation; (**b**) xz-Variation; (**c**) yz-Variation.

The results of the analysis of accuracy *Acc* and repeatability *Rep* (*cf*. Section 3.3) of the Leap Motion controller by measuring the reference pen tip position in a static scenario, thus when the robot stays in a desired static position (in each case *R* = 5, 000 measurements), are illustrated in Figure 4. The particular diagrams visualize the deviation between the desired static Cartesian position and the measured positions relative to the xy-plane (a), xz-plane (b) and yz-plane (c). Independent from the axis, the deviation between the desired position and the measured positions is less than 0.20 mm. In the case of the x-axis the deviation is less than 0.17 mm.

**Figure 5.**
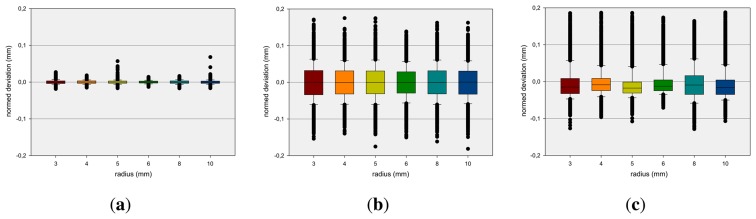
Box-and-whisker Plots for different Tool Diameters: *d* = 3 mm, *d* = 4 mm, *d* = 5 mm, *d* = 6 mm, *d* = 8 mm, *d* = 10 mm of the average Deviation concerning (**a**) x-; (**b**) y- and (**c**) z-Axis.

A relevant question is if the dimension of the reference pen influences the measurement results. This is analyzed by measuring static positions of pen tips with different diameters. The box-and-whisker plots [24] in Figure 5 show the average deviation concerning the x-, y- and z-axis at different diameters (see Section 4) of the reference pen. The boxes are representing the interquartile range that contains 50% of the values and the whiskers are marking the minimum and maximum values, excluding outliers (marked as black points). The mean x-deviation is approximately 0 mm while mean y- and z-deviations show higher variances. It can be observed that the measurements are independent from the radius of the reference pen.

Table 1 shows a comparison of the repeatability values *Rep* (*cf*. Equation (3)) of tools with varying diameters. The corresponding variances are below 0.065 mm. The standard deviations are below 0.05 mm. No significant correlation between a tool's diameter and the corresponding repeatability is observed. Only a weak anti-correlation with a correlation coefficient (Pearson) of −0.4328 and statistical significance of 0.391 is detected. Hence the mean repeatability value of the Leap Motion controller of 0.2 mm can be obtained from this measurement setup.

**Figure 6.**
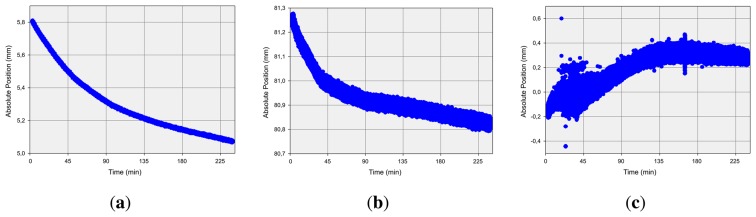
Long Time Measurement: Change of (**a**) x-, (**b**) y- and (**c**) z-Coordinate over Time.

Furthermore, the effect of continuous operation on the measurement quality was analyzed. Thus a long time measurement of 240 minutes of a static reference pen tip position was performed. The results are illustrated in Figure 6 as a function of the particular Cartesian values of the x-, y- and z-axis over the time respectively. As can be observed, the deviation of the measured values is below 0.8 mm, independent from the axis. This is equivalent to a motion of less than 1.4 mm in 240 minutes, which is less than one-thousandth of a micrometer per second.

### 5.2. Dynamic Test Cases

**Figure 7.**
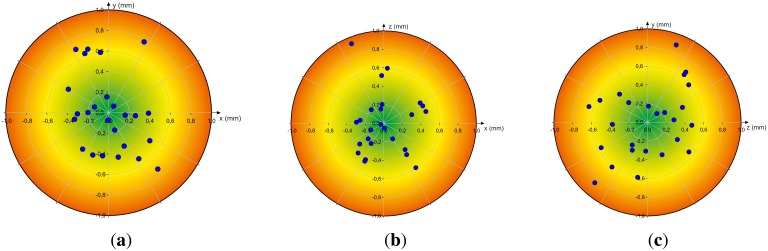
Analysis of Accuracy: Deviation between a Desired 3D Position and the Median of the Measured Positions, (**a**) xy-Plane; (**b**) xz-Plane; (**c**) yz-Plane.

**Figure 8.**
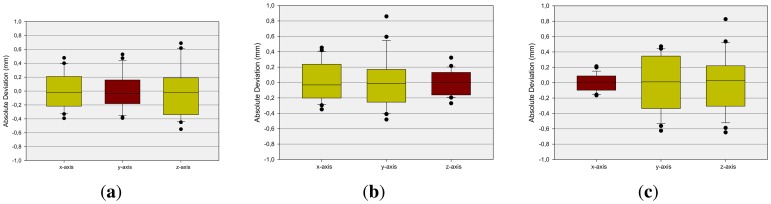
Analysis of Accuracy of the Measured Positions. The fixed Orientations are marked in red. (**a**) xy-Plane; (**b**) xz-Plane; (**c**) yz-Plane.

Next, the dynamic scenarios are analyzed by positioning the reference pen tip in different positions using the robot. Figure 7 illustrates the analysis of the accuracy when positioning the reference tip on different positions on a regular grid in the xy-, xz- and yz-plane by the robot. The diagrams show the deviation between a desired 3D position and the median of the corresponding measured positions respectively. Independent from the axis the deviation is below 1mm and on average under 0.4 mm as illustrated in Figure 8 in terms of box-whisker-plots. This has a high relevance in terms of proper sensor-based user-interface design.

**Figure 9.**
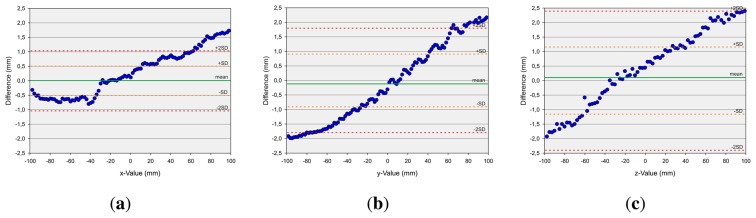
Analysis of Accuracy as Bland-Altman Plots for the three axis-aligned movements of the Test Bar of the Robot for a measuring range from −100 mm to 100 mm. (**a**) x-axis; (**b**) y-axis; (**c**) z-axis.

**Figure 10.**
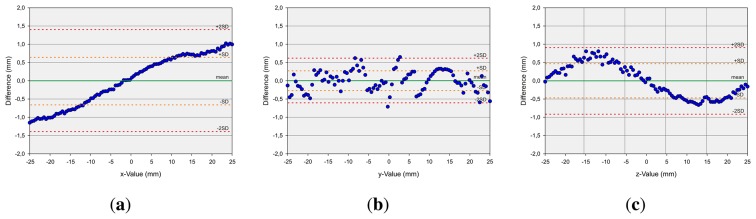
Analysis of Accuracy as Bland-Altman Plots for a Sinus Shaped Motion of the Test Bar of the Robot for a measuring range from − 25 mm to 25 mm. (**a**) x-Deviation; (**b**) y-Deviation; (**c**) z-Deviation.

The second measurement setup, in the case of dynamic pen tip positioning, refers to the measurement of discrete points on a robot path. First axis-aligned movements are evaluated. Linear paths of 200 mm along the xy-, xz- and yz-planes, centered on the center point of the Leap Motion controller, are generated and drawn by the robot. Figure 9 shows the evaluation of these measurements with Bland-Altman diagrams [25]. The graph represents the mean difference (green line) of the position of the robot and the measured value by the Leap Motion controller (once the robot has arrived at its target position) and limits of agreement as the mean difference (*mean*)—orange (±*sd*) and red (±2*sd*) dotted lines. As can be observed, the absolute values of the mean deviations are in all cases less than 0.1 mm, while the x-deviation is approximately 0 mm. The standard deviation is approximately 0.5 mm in the case of the x-values, above 1.0 mm in the case of the y-values and below 1.0 mm in the case of the z-values. The x- and y-values show a statistically significant correlation with a correlation coefficient (Pearson) of 0.875. The x- and z-values have even more significant correlation with a correlation coefficient of 0.979, and the y- and z-values show significant correlation with a correlation coefficient of 0.955. It can be seen that the deviation in the sensor's position detection accuracy is a monotonic function of the distance to the origin of the Leap Motion controller's coordinate system. It can be stated that the Leap Motion controller is more accurate on the x-dimension. On the y- and z-dimension, less accurate results are obtained. It seems that this is caused by the horizontal alignment of the IR cameras. Subsequently, a path along a sinus function in the xy-plane is drawn with a support of 50 mm again centered on the controller's center point. This relatively small support is chosen in order to minimize the detected distortions based on the distance of the desired point to the controller's center point. As can be seen in Figure 10, the mean deviation is in all cases approximately 0 mm. The standard deviation *sd* is below 0.7 mm in the case of the x-values, below 0.5 mm in the case of the y-values and below 0.3 mm in the case of the z-values. Overall, the evaluation of the dynamic scenarios shows the high potential of the Leap Motion controller in gesture detection systems, where accurate path determination is of superior importance.

## 6. Discussion and Conclusions

In this paper, a first study of the Leap Motion controller in the preliminary version was presented. The Leap Motion controller introduces a new gesture and position tracking system with sub-millimeter accuracy. Taking into account that the position determined by the sensor has a direct influence on the quality of the gesture recognition, the main focus of attention was on the evaluation of the accuracy and repeatability. The analysis was performed in accordance to ISO 9283. In consideration of the fact that the accuracy attainable by the human hand is on average around 0.4 mm, an accurate experimental setup was drafted accordingly. This consists of an industrial robot with a reference pen that allows a position accuracy of 0.2 mm.

First, the measurement accuracy for static setups was analyzed. Thereby an axis-independent deviation between a desired 3D position and the measured positions less than 0.2 mm has been obtained. Furthermore, there was no observable influence of the radius of the reference pen upon the accuracy. Secondly, an evaluation for dynamic situations was performed, *i.e.*, the tip of the robot was moved to different coordinate positions on a regular grid on a plane as well as to discrete positions on different paths (Section 5.2). Independent from the plane, an accuracy of less than 2.5 mm could be obtained (average of 1.2 mm). The repeatability had an average of less than 0.17 mm. When moving to discrete positions on a path, the standard deviation was below 0.7 mm per axis. It can be summarized that it was not possible to achieve the theoretical accuracy of 0.01 mm under real conditions but a high precision (an overall average accuracy of 0.7mm) with regard to gesture-based user interfaces. Comparable controllers in the same price range, e.g., the Microsoft Kinect, were not able to achieve this accuracy. It should be mentioned that the presented evaluation results were executed on the preliminary version of the Leap Motion controller and can differ from the accuracy and repeatability of the consumer product.

Future work will focus, in particular, on questions considering the utilization of the Leap Motion controller in the field of hand gesture recognition, especially for the design of user interfaces. A first approach could be the analysis of the performance using Fitts' law [26]. Further possibilities are the integration of the Leap Motion controller into many current applications in diverse fields, for example an interactive navigation tool for Medical Volume Data (e.g., for OsiriX (OsiriX, http://www.osirix-viewer.com)) or a direct 3D modelling tool (e.g., for Autodesk 3ds Max (Autodesk 3ds Max, http://www.autodesk.com/3ds-max)). Beyond that, the Leap Motion provides innovation potential for the development of many new applications.

The presented robot-based evaluation setup is not limited to the presented use case of analyzing the Leap Motion controller. The modified robot cell will be utilized to evaluate future motion detection or position tracking systems.

## Figures and Tables

**Table 1.** Comparison of the Repeatability of Tools with varying Diameters.

**Diameter (mm)**	**Repeatability (mm)**	**Variance (mm)**	**Standard Deviation (mm)**
3.0	0.2056	0.0620	0.0479
4.0	0.1694	0.0541	0.0384
5.0	0.1304	0.0458	0.0285
6.0	0.1672	0.0513	0.0386
8.0	0.1964	0.0626	0.0446
10.0	0.1276	0.0473	0.0268
